# Drug-target interaction prediction using Multi Graph Regularized Nuclear Norm Minimization

**DOI:** 10.1371/journal.pone.0226484

**Published:** 2020-01-16

**Authors:** Aanchal Mongia, Angshul Majumdar

**Affiliations:** 1 Dept. of Computer Science and Engineering, IIIT-Delhi, Delhi, India; 2 Dept. of Electronics and Communications Engineering, IIIT-Delhi, Delhi, India; Liverpool John Moores University, UNITED KINGDOM

## Abstract

The identification of potential interactions between drugs and target proteins is crucial in pharmaceutical sciences. The experimental validation of interactions in genomic drug discovery is laborious and expensive; hence, there is a need for efficient and accurate in-silico techniques which can predict potential drug-target interactions to narrow down the search space for experimental verification. In this work, we propose a new framework, namely, Multi-Graph Regularized Nuclear Norm Minimization, which predicts the interactions between drugs and target proteins from three inputs: known drug-target interaction network, similarities over drugs and those over targets. The proposed method focuses on finding a low-rank interaction matrix that is structured by the proximities of drugs and targets encoded by graphs. Previous works on Drug Target Interaction (DTI) prediction have shown that incorporating drug and target similarities helps in learning the data manifold better by preserving the local geometries of the original data. But, there is no clear consensus on which kind and what combination of similarities would best assist the prediction task. Hence, we propose to use various multiple drug-drug similarities and target-target similarities as multiple graph Laplacian (over drugs/targets) regularization terms to capture the proximities exhaustively. Extensive cross-validation experiments on four benchmark datasets using standard evaluation metrics (AUPR and AUC) show that the proposed algorithm improves the predictive performance and outperforms recent state-of-the-art computational methods by a large margin. Software is publicly available at https://github.com/aanchalMongia/MGRNNMforDTI.

## Introduction

The field of drug discovery in Pharmaceutical Sciences is plagued with the problem of high attrition rate. The task is to find effective interactions between chemical compounds (drugs) and amino-acid sequences/ proteins (targets). This is traditionally done through wet-lab experiments which are costly and laborious.

An effective and appropriate alternative to avoid costly failures is to computationally predict the interaction probability. A lot of algorithms have been proposed for DTI (Drug-target interaction) prediction in recent years [1, 2], which use small number of experimentally validated interactions in existing databases such as ChEMBL [3], DrugBank [4], KEGG DRUG [5], STITCH [6] and SuperTarget [7]. Identification of drug-target pairs leads to improvements in different research areas such as drug discovery, drug repositioning, polypharmacology, drug resistance and side-effect prediction [8].

For example, Drug repositioning [9, 10] (reuse of existing drugs for new indications) can grant polypharmacology (multi-target effect) to a drug. One of the many successfully repositioned drugs is Gleevec (imatinib mesylate). Earlier, it was known to interact only with the Bcr-Abl fusion gene which is indicative of leukemia. However, later discoveries showing that it also interacts with PDGF and KIT, repositioned it for the treatment of gastrointestinal stromal tumors [11, 12].

There are three major classes of computational methods for predicting DTI: Ligand-based, Docking based, and Chemogenomic approaches. Ligand-based approaches leverage the similarity between target proteins’ ligands to predict interactions [13]. The idea is that molecules with similar structure/property would bind similar proteins [14]. But, the reliability of results might get compromised due to limited information about known ligands per protein. Docking-based approaches use the 3D structure of both drugs and proteins to predict the interaction likelihood [15–17]. This, although is well-accepted, but is very time-consuming and hence, cannot be used for protein families for which the 3D structure is either difficult to predict or is unavailable [18] like the G-protein coupled receptors (GPCRs).

Chemogenomic approaches overcome the challenges of traditional methods and thus, have recently gained much attention. The approaches under this category can work on the huge amount of biological data, publicly available in existing online databases and can process metadata (chemical structures and genomic sequences) for both the drug and target, respectively. These approaches can further sub-classified based on the representation of the input data: Feature-based techniques and Similarity-based techniques. Feature-based techniques take their inputs in the form of features and class labels (binary values here) and leverage machine learning for classifying if an input instance corresponds to a positive interaction or a negative one. Examples of typical feature based methods include Decision Trees (DT), Random Forests (RF) and Support Vector Machines (SVM) to build classification models based on the labeled feature vectors [19]. In the training set, positive instances are the experimentally known interactions while the negative ones are either non-interactions or unknown interactions. The other category of chemogenomic techniques, Similarity-based methods, use two similarity matrices corresponding to drug and target similarity, respectively, along with the drug-target interaction matrix.

Let us discuss the similarity between the said DTI problem and the problem of collaborative filtering (CF). CF is a standard problem in information retrieval. It is used in recommendations systems (e.g. in Netflix movie recommendations and Amazon product recommendations). There is a database of user’s and their ratings on items (movies, products, etc.). Obviously, not all the ratings are available; users typically rate only a small subset of items. The objective is to estimate the ratings of all the users on all the items. If that can be done accurately, recommendation accuracy increases. The similarity between DTI and CF should be straightforward now; the drugs play the role of users and the targets play the role of items. The interactions are similar to the ratings. Over the years, many approaches originally developed for CF have been leveraged to solve the DTI problems.

In both CF [20] and DTI [21–23], the initial techniques were based on simple neighborhood-based models. In order to predict the interaction of a (active) drug on a target, the first step is to find out similar (neighbor) drugs by computing some kind of a similarity score. Once the neighborhood is obtained, the interaction value from the drugs in the neighborhood are weighted (by the normalized similarity score) to interpolate the interaction of the active drug on the target. This is similar to KNN (K-nearest neighbor) based approaches in DTI prediction literature [24]. Another approach was based on bipartite local models. In such models, a local model is built for every drug and target. For example in [25] an SVM was trained for each to predict the interaction of each drug on all targets and each target on all drugs. Finally, the decision from the two was fused. This is just an example, there are other techniques falling under this generic approach like [26, 27]. The above-mentioned methods can be categorized as classification based approaches where the chemical/biological information is used to generate features for drugs and targets individually and these two types of features are then concatenated and the corresponding interaction is assumed to the class corresponding to this feature. Any standard classifier is generally used for the final classification. In such class of techniques, the emphasis is on different feature selection mechanisms [28, 29]. Both semi-supervised [30, 31] and supervised [32, 33] classification based prediction approaches have been leveraged in Drug-Target interaction prediction.

The second category is based on network diffusion models. One technique for DTI prediction based on such models is based on a random walk on the network with a predefined transition matrix [34]. Another work falling under this category, predicts interactions by finding a simple path (without loops) between nodes of the network.

The third approach is based on matrix factorization. These techniques were originally developed for collaborative filtering [35]. It is assumed that drugs and targets are characterized by latent factors. The probability of interaction is high when the latent factors match; i.e. when the inner product has a high value. Therefore, it is logical to express the interaction matrix as a (an inner) product of drug and target latent factors. This allows matrix factorization (and its variants) to be applied [36, 37].

In a very recent review paper [2] it was empirically shown that matrix factorization based techniques yields by far the best results. The fundamental assumption behind matrix factorization to work is that there are very few (latent) factors that are responsible for drug target interactions. This is the reason, one can factor the DTI matrix into a tall (drug) latent factor matrix and a fat (target) latent factor matrix. Mathematically speaking, the assumption is that the DTI matrix is of low-rank. Matrix factorization is being used to model low-rank matrices for the past two decades since the publication of Lee and Seung’s seminal paper [38]. However, matrix factorization is a bi-linear non-convex problem; there are no convergence guarantees. In order to ameliorate this problem, mathematicians proposed an alternative approach based on nuclear norm minimization [39–41]. The nuclear norm is the closest convex surrogate to the rank minimization (known to be NP-hard) problem and there are provable mathematical guarantees on its equivalence to rank minimization.

The standard versions of both the matrix factorization and nuclear norm minimization techniques are unable to incorporate similarity information of the drugs and the targets. In recent studies [37, 42], it was shown that the best results are obtained when these technique incorporate graph regularization penalties into them. But, these works regularize the objective function by taking into account, just the standard chemical structure similarity for drugs (*S*_*d*_) and the genomic sequence similarity for targets (*S*_*t*_). No study in literature gives a clear picture of which kind of similarities would be the best for DTI prediction. We, therefore, incorporate different other kinds of similarities and a combination of them as a multi-graph Laplacian regularization with Nuclear Norm Minimization for DTI prediction. The algorithm uses four new similarity measures over the drugs and targets, apart from the standard similarities to construct the graph Laplacians. The four newly incorporated similarities are computed from the interaction matrix and take into account the Cosine similarity, Correlation, Hamming distance and Jaccard similarity between the drugs and targets. To the best of our knowledge, this is the first work on multiple graph laplacian regularized nuclear norm minimization for DTI prediction.

## Materials and methods

### Dataset description

We use the four benchmark datasets introduced in [21] having four different classes of proteins: enzymes (Es), ion channels (ICs), G protein- coupled receptors (GPCRs) and nuclear receptors (NRs). The data was simulated from public databases KEGG BRITE [43], BRENDA [44] SuperTarget [7] and DrugBank [4] and is publically available at the given link: http://web.kuicr.kyoto-u.ac.jp/supp/yoshi/drugtarget/.

The data from each of these databases is formatted as an adjacency matrix, called interaction matrix between drugs and targets, encoding the interaction between n drugs and m targets as 1 if the drug *d*_*i*_ and target *t*_*j*_ are known to interact and 0, otherwise.

Along with the interaction matrix, drug similarity matrix *S*_*d*_ and a target similarity matrix *S*_*t*_ are also provided. In *S*_*d*_, each entry represents the pairwise similarity between the drugs and is measured using SIMCOMP [45]. It represents the chemical structure similarity between drugs; measured using the number of shared substructures within the chemical structures of two drugs. In *S*_*t*_, the similarity score between two proteins is the genomic sequence similarity. It is based on the amino acid sequences of the target protein and is computed using normalized Smith–Waterman [46].

The similarity matrices *S*_*d*_ and *S*_*t*_ constitute the most standard similarities that have been used in the DTI prediction task hitherto. We use these similarities along with the following four more similarities computationally derived from the drug-target interaction matrix to form the graph laplacian terms:

- Cosine similarity: measures the cosine of the angle between two drug/target vectors projected in a multi-dimensional space. Its value ranges from -1 (exactly opposite) to 1 (exactly the same). Given two *n*-dimensional drug (or target) vectors (A and B), the cosine similarity is calculated as follows:
  $$\begin{array}{c} S^{\text{cos}}=\frac{\sum_{i=1}^{n}A_{i}B_{i}}{\sqrt{\sum_{i=1}^{n}A_{i}^{2}}\sqrt{\sum_{i=1}^{n}B_{i}^{2}}} \end{array}$$
  Here, *A*_*i*_ and *B*_*i*_ denote the components of vector A and B.
- Correlation: computes the Pearson’s linear correlation coefficient indicating the extent to which two variables are linearly related. It has a value between +1 and −1, where 1 is total positive linear correlation, 0 is no linear correlation, and −1 is total negative linear correlation. For a pair (say A and B) of drugs/targets with sample size *n*, it is given by:
  $$\begin{array}{c} S^{cor}=\frac{\sum_{i=1}^{n}\left(A_{i}-\bar{A}\right)\left(B_{i}-\bar{B}\right)}{\sqrt{\sum_{i=1}^{n}{\left(A_{i}-\bar{A}\right)}^{2}}\sqrt{\sum_{i=1}^{n}{\left(B_{i}-\bar{B}\right)}^{2}}} \end{array}$$
  where
  $$\begin{array}{c} \bar{A}=\frac{1}{n}\sum_{i=1}^{n}A_{i}\;\text{and}\;\bar{B}=\frac{1}{n}\sum_{i=1}^{n}B_{i} \end{array}$$
- Hamming similarity: has been computed using Hamming distance. For any two *n*-dimensional drugs/targets (A and B), the hamming distance is the percentage of interaction positions that differ. We calculate Hamming distance based similarity by simply subtracting hamming distance from 1, giving us its complementary (the percentage of common interaction positions for a pair of drugs/targets). It can be calculated as follows:
  $$\begin{array}{c} S^{ham}=1-\frac{\#(A_{i}\neq B_{i})}{n} \end{array}$$
- Jaccard similarity: is defined as the percentage of common non-zero interaction positions for the two given sample sets of drugs/target vectors.
  $$\begin{array}{c} S^{jac}=\frac{\#[\left(A_{i}=B_{i}\right)\cap \left(\left(A_{i}\neq 0\right)\cup \left(B_{i}\neq 0\right)\right)]}{\#[\left(A_{i}\neq 0\right)\cup \left(B_{i}\neq 0\right)]} \end{array}$$

Table 1 summarizes the statistics of all four datasets.

**Table 1:** Drugs, targets and interactions in each dataset used for validation.

Datasets	Nuclear Receptor (NR)	G-Protein Coupled Receptor (GPCR)	Ion channel (IC)	Enzyme (E)
# Interactions	90	635	1476	2926
# Drugs	54	223	210	445
# Targets	26	95	204	664

### Nuclear norm minimization

Let us assume that *X* is the adjacency matrix where each entry denotes interaction between a drug and target (1 if they interact, 0 otherwise). Unfortunately, we only observe this matrix partially because all interactions are not known. If *M* denotes the partially observed adjacency matrix, the mathematical relation between *X* and *M* is expressed as:

$$\begin{array}{c} M=A(X) \end{array}$$ (1)

In the above equation, *A* denoted the sub-sampling operator, element-wise multiplied to *X*. It is nothing but a binary matrix or a mask that has 0’s where the interaction *X* has not been observed or is unknown and 1’s where they have been. The task is to recover *X*, given the observations *M*, and the sub-sampling mask *A*. It is known that *X* is of low-rank. Ideally, X should be recovered by (2).

$$\begin{array}{c} \underset{X}{\text{min}}\;rank\left(X\right)\;\text{such}\;\text{that}\;M=A\left(X\right) \end{array}$$ (2)

Unfortunately, rank minimization is an NP-hard problem with doubly exponential complexity, therefore solving it directly is not feasible.

Traditionally, a low-rank matrix has been modeled as a product of a thin and a fat matrix and recovered using Matrix Factorization techniques [38]. But, Matrix Factorization is a bi-linear non-convex problem, therefore there is no guarantee for global convergence. In the past decade, mathematicians showed that the rank minimization problem can be relaxed by its convex surrogate (nuclear norm minimization) with provable guarantees [39, 40] This turns out to be a convex problem that can be solved by Semi-Definite Programming. More efficient solvers have also been proposed. Problem (2) is expressed as (3)

$$\begin{array}{c} \underset{X}{\text{min}}\;\|X{\|}_{*}\;\text{such}\;\text{that}\;M=A\left(X\right) \end{array}$$ (3)

Here the nuclear norm (|| ||_*_) is defined as the sum of singular values of data matrix *X*. It is the *l*_1_ norm (sum of absolute values) of the singular values of *X* and is the tightest convex relaxation of the rank of the matrix, and hence its ideal replacement.

Here, (3) is a constrained formulation for the noiseless scenario, usually its relaxed version, (4) is solved.

$$\begin{array}{c} \underset{X}{\text{min}}\;\|M-{A\left(X\right)\|}_{F}^{2}+\lambda \|X{\|}_{*} \end{array}$$ (4)

One of the efficient solvers for Nuclear Norm minimization is the Singular value shrinkage (SVS) algorithm [47].

**Algorithm 1** Singular value shrinkage

1: **procedure** Matrix-SVS(*M*, *A*, λ)

2:  **Initialize**: *X* = *rand*, *a*

3:   **For loop**, iterate (k)

4:    $B_{k}=X_{k-1}+\frac{1}{a}A^{T}\left(M-A\;{}^{\circ}\;X_{k-1}\right)$

5:    Compute SVD of *B*: *B*_*k*_ = *USV*^*T*^

6:    Soft threshold the singular values: Σ = *soft*(*S*, λ/2)

7:    *X*_*k*_ = *U*Σ*V*^*T*^

8:    $X_{k}\leftarrow X_{k}^{+}$

9:  **End loop 1**

### Multi Graph Regularized Nuclear Norm Minimization

Nuclear Norm based Low-rank Matrix Completion is not our contribution, it has been around since the past decade. The problem with standard Nuclear norm minimization (NNM) is that it cannot accommodate associated information such as Similarity matrices for Drugs and Targets. But, it has been seen in recent studies that accommodating the similarity information is crucial for improving the DTI prediction results. The current works have incorporated the standard similarity measures for drugs and targets in matrix factorization [37] and Matrix completion [42] frameworks. It is imperative that NNM should be capable of taking into account more types and combinations of similarities. To achieve this, we have augmented four other types of similarities between drugs/targets and presented Multi-Graph regularized Nuclear Norm Minimization (MGRNNM).

Graph regularization assumes that data points which are in the neighborhood of each other in the original space should also be close to each other in the learned manifold (**Local Invariance assumption**). So, Graph regularization would allow/enable the algorithm to learn manifolds for the drug and target spaces in which the data is assumed to lie. The multi graph regularized version of Nuclear norm minimization, aims to prevent over fitting and greatly enhance the generalizing capabilities. It is incorporated into the formulation/objective function as Laplacian weights corresponding to drugs and targets:

$$\begin{array}{c} \underset{X}{\text{min}}\;\|M-{A\left(X\right)\|}_{F}^{2}+\lambda \|X{\|}_{*}+\mu_{1}Tr\left(X^{T}\sum_{i=1}^{nos}L_{d}^{i}X\right)+\mu_{2}Tr\left(X\sum_{i=1}^{nos}L_{t}^{i}X^{T}\right) \end{array}$$ (5)

where λ ≥ 0, *μ*_1_ ≥ 0 and *μ*_2_ ≥ 0 are parameters balancing the reconstruction error of NNM in the first two terms and graph regularization in the last two terms, *Tr*(.) denotes the trace of the matrix, *nos* stands for number of similarity matrices (*nos* = 5 in our case).

If, say we consider a single similarity matrix for drugs (*S*_*d*_) and that for targets (*S*_*t*_), then *L_d_* = *D_d_* − *S_d_* and *L_t_* = *D_t_* − *S_t_* are the graph Laplacians [48] for *S*_*d*_ (drug similarity matrix) and *S*_*t*_ (target similarity matrix), respectively, and $D_{d}^{ii}=\Sigma_{j}S_{d}^{ij}$ and $D_{t}^{ii}=\Sigma_{j}S_{t}^{ij}$ are degree matrices.

Problem (5) is solved using a variable splitting approach [49]. The augmented Lagrangian is expressed as (6). We introduce two new proxy variables *Z* and *Y* such that *Z*^*T*^ = *X* and *Y* = *X*.

$$\begin{array}{c} \begin{array}{c} \underset{X,Y,Z}{\text{min}}\;\|M-{A\left(X\right)\|}_{F}^{2}+\lambda \|X{\|}_{*}+\mu_{1}Tr\left(Z\sum_{i=1}^{nos}L_{d}^{i}Z^{T}\right)+\mu_{2}Tr\left(Y\sum_{i=1}^{nos}L_{t}^{i}Y^{T}\right)+\\ \nu_{1}\|Z^{T}-{X\|}_{F}^{2}+\nu_{2}\|Y-{X\|}_{F}^{2} \end{array} \end{array}$$ (6)

The variables are updated using ADMM [50, 51]. This leads to the following subproblems (7), (8) and (9)

$$\begin{array}{c} X\leftarrow \underset{X}{\text{min}}\;\|M-{A\left(X\right)\|}_{F}^{2}+\nu_{1}\|Z^{T}-{X\|}_{F}^{2}+\nu_{2}\|Y-{X\|}_{F}^{2}+\lambda \|X{\|}_{*} \end{array}$$ (7)

$$\begin{array}{c} Y\leftarrow \underset{Y}{\text{min}}\;\mu_{2}Tr\left(Y\sum_{i=1}^{nos}L_{t}^{i}Y^{T}\right)+\nu_{2}\|Y-{X\|}_{F}^{2} \end{array}$$ (8)

$$\begin{array}{c} Z\leftarrow \underset{Z}{\text{min}}\;\mu_{1}Tr\left(Z\sum_{i=1}^{nos}L_{d}^{i}Z^{T}\right)+\nu_{1}\|Z^{T}-X{\|}_{F}^{2} \end{array}$$ (9)

Problem (7) can be expressed as a standard NNM probelm (by column stacking the variables).

$$\begin{array}{c} {\left\|(\begin{array}{c} M\\ \sqrt{\nu_{1}}Z^{T}\\ \sqrt{\nu_{2}}Y \end{array})-(\begin{array}{c} A\\ \sqrt{\nu_{1}}I\\ \sqrt{\nu_{2}}I \end{array})X\right\|}_{F}^{2}+\lambda \|X{\|}_{*} \end{array}$$ (10)

To solve for *Y* and *Z*, we differentiate (8) and (9) wrt *Y* and *Z*, respectiveley.

$$\begin{array}{c} Y=\underset{Y}{\text{arg}\;\text{min}}\left(F_{1}\right)\;\text{where}\;F_{1}=\mu_{2}Tr\left(Y\sum_{i=1}^{nos}L_{t}^{i}Y^{T}\right)+\nu_{2}\|Y-{X\|}_{F}^{2} \end{array}$$ (11)

$$\begin{array}{c} Z=\underset{Z}{\text{arg}\;\text{min}}\left(F_{2}\right)\;\text{where}\;F_{2}=\mu_{1}Tr\left(Z\sum_{i=1}^{nos}L_{d}^{i}Z^{T}\right)+\nu_{1}\|Z^{T}-X{\|}_{F}^{2} \end{array}$$ (12)

$$\begin{array}{c} \frac{\partial F_{1}}{\partial Y}=\mu_{2}\left(Y{\left(\sum_{i=1}^{nos}L_{t}^{i}\right)}^{T}+Y\sum_{i=1}^{nos}L_{t}^{i}\right)+2\nu_{2}\left(Y-X\right) \end{array}$$ (13)

$$\begin{array}{c} \frac{\partial F_{1}}{\partial Y}=\mu_{2}Y\left[\sum_{i=1}^{nos}\left({L_{t}^{i}}^{T}+L_{t}^{i}\right)\right]+2\nu_{2}\left(Y-X\right) \end{array}$$ (14)

Since *L*_*t*_ is a symmetric matrix, $L_{t}^{T}=L_{t}$. So,

$$\begin{array}{c} \frac{\partial F_{1}}{\partial Y}=2\mu_{2}Y\sum_{i=1}^{nos}L_{t}^{i}+2\nu_{2}\left(Y-X\right) \end{array}$$

Equating the derivative to zero, we get:

$$\begin{array}{c} \nu_{2}Y+\mu_{2}Y\sum_{i=1}^{nos}L_{t}^{i}=\nu_{2}X \end{array}$$ (15)

The matrix equation of this form (AT+TB = C) cannot be solved directly for variable T and is called Sylvester equation. Such an equation has a unique solution when the eigenvalues of A and -B are distinct.

A similar Sylvester equation and update step for *Z* can be obtained by differentiating *F*_2_ and equating to 0.

$$\begin{array}{c} \nu_{1}Z+\mu_{1}Z\sum_{i=1}^{nos}L_{d}^{i}=\nu_{1}X^{T} \end{array}$$ (16)

It can be shown that computing the sum of the Graph Laplacians is equivalent to computing the Laplacian from the sum of various similarity matrices involved. For instance, consider the sum of drug Graph Laplacians:

$$\begin{array}{c} \begin{array}{c} \sum_{i=1}^{nos}L_{d}^{i}\\ =\sum_{i=1}^{nos}\left(D_{d}^{i}-S_{d}^{i}\right)\\ =\sum_{i=1}^{nos}D_{d}^{i}-\sum_{i=1}^{nos}S_{d}^{i}\\ =\sum_{i=1}^{nos}diag(\sum_{j}^{}S_{d}^{j})-\sum_{i=1}^{nos}S_{d}^{i}\\ =diag\left(\sum_{j}^{}(\sum_{i=1}^{n}S_{d}^{i}{)}^{j}\right)-\sum_{i=1}^{nos}S_{d}^{i} \end{array} \end{array}$$

Let $\sum_{i=1}^{nos}S_{d}^{i}=S_{d}^{COM}$ where $S_{d}^{COM}$ stands for combined similarity for drugs. Essentially,

$$\begin{array}{c} S_{d}^{COM}=S_{d}+S_{d}^{cos}+S_{d}^{cor}+S_{d}^{ham}+S_{d}^{jac} \end{array}$$ (17)

łThen,

$$\begin{array}{c} \sum_{i=1}^{nos}L_{d}^{i}=diag\left(\sum_{j}^{}S_{d}^{COM}\right)-S_{d}^{COM}=D_{d}^{COM}-S_{d}^{COM}=L_{d}^{COM} \end{array}$$ (18)

Here, $D_{d}^{COM}$ and $L_{d}^{COM}$ denote combined degree matrix and combined Laplacian matrix (sum of graph laplacians) for drugs. Of note, the individual Laplacians or the similarities can be weighted unequally to give more or less emphasis on a specific type of similarity. The pseudo-code for MGRNNM has been given in Algorithm 2.

**Fig 1:**
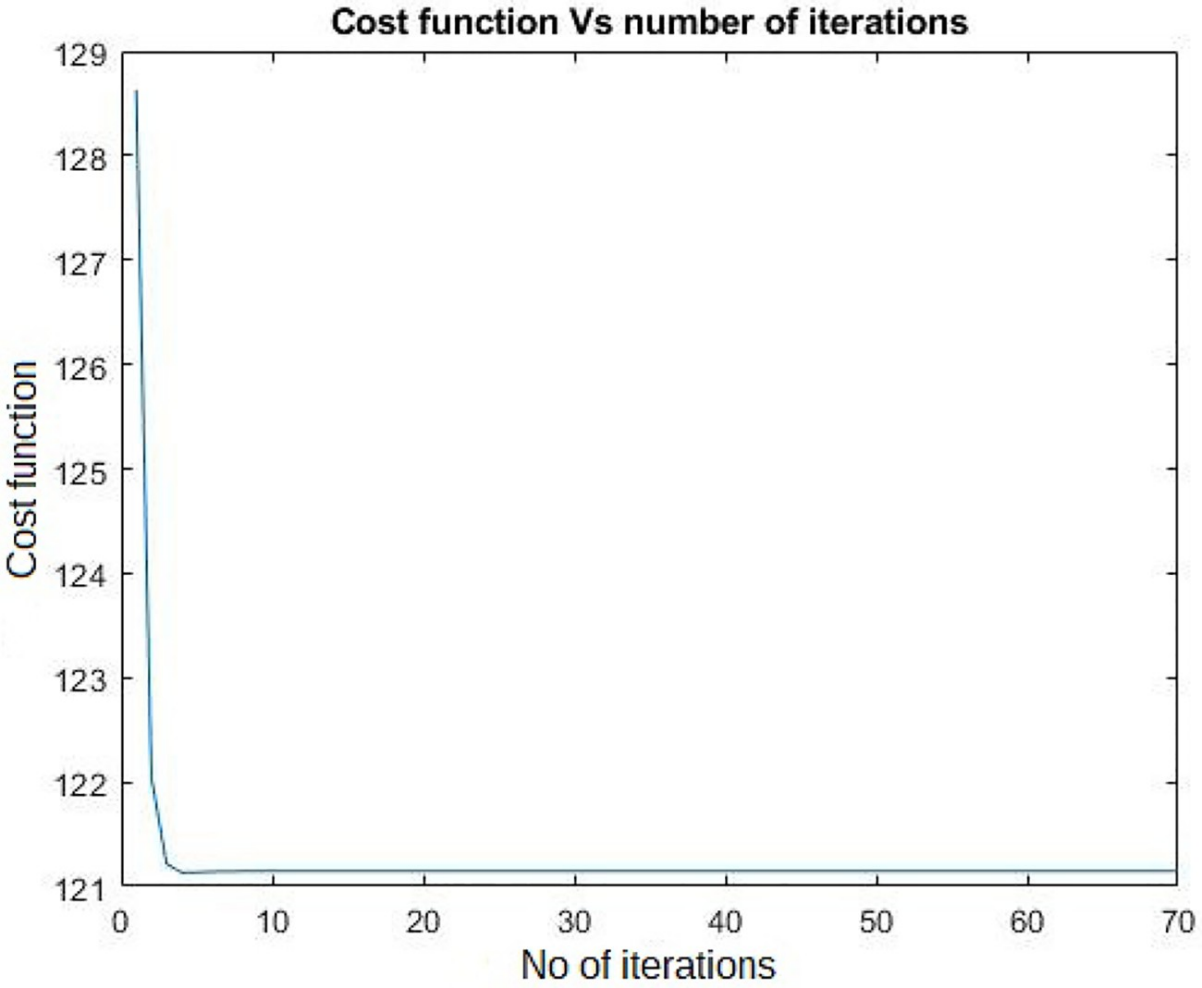
Converge plot of the MGRNNM algorithm for NR dataset with cross validation setting CVS1 (drug-target pair prediction).

The standard NNM is a convex problem and the introduced graph regularization penalties are also convex, so entire formulation (5), being a sum of convex functions, is convex. Therefore it is bound to converge to a global minima. We chose the number of iterations such that the algorithm halts when the objective function does not change with iterations. A sample convergence plot for one of the datasets for drug-target pair prediction has been shown in Fig 1.

**Algorithm 2** Multi Graph regularized Nuclear Norm Minimization

1: **procedure** MGRNNM($M,A,S_{d}^{COM},S_{t}^{COM}$)

2:  **Sparsify**: $S_{d}^{COM},S_{t}^{COM}$

3:  **Initialize**: $\lambda ,\mu_{1},\mu_{2},\nu_{1},\nu_{2},L_{d}^{COM},L_{t}^{COM},Y=M,Z=M^{T}$

4:    $AA\leftarrow (\begin{array}{c} A\\ \sqrt{\nu_{1}}I\\ \sqrt{\nu_{2}}I \end{array})$

5:   **For loop**, iterate (k)

6:    $YY_{k}\leftarrow (\begin{array}{c} M\\ \sqrt{\nu_{1}}Z^{T}\\ \sqrt{\nu_{2}}Y \end{array})$

7:    *X*_*k*_ ← *MATRIX* − *SVS*(*YY*_*k*_, *AA*, λ)

8:    $Y_{k}\leftarrow \text{solve-sylvester}\left(\nu_{1}I,\mu_{1}L_{d}^{COM},\nu_{1}X_{k}^{\prime }\right)$

9:    $Z_{k}\leftarrow \text{solve-sylvester}\left(\nu_{2}I,\mu_{2}L_{t}^{COM},\nu_{2}X_{k}\right)$

10:  **End loop 1**

### Time complexity of MGRNNM

The algorithm is iterative, so we only discuss the time complexity per iteration. In each iteration, we solve the two Sylvester equations and one NNM. NNM itself is an iterative algorithm that requires solving Singular value decomposition in each iteration, the order of complexity of which is *O*(*n*^3^). The complexity of solving Sylvester equation is *O*(*n*^*b*^.*log*(*n*)) [52] where *b* is between 2 and 3.

## Results and discussion

### Experimental setup

We validated our proposed method by comparing it with recent and well-performing prediction methods proposed in the literature. Out of the 7 approaches with which we compare,

- Five are specifically designed for DTI task (WGRMF: Weighted Graph Regularized Matrix Factorization, CMF: Collaborative Matrix Factorization, RLS_WNN: Regularized Least square Nearest neighbor profile, NRLMF: Neighborhood Regularized Logistic Matrix Factorization for Drug-Target Interaction Prediction and TMF: Triple matrix factorization) [23, 37, 53–55]. Of note, the code available for TMF does not reproduce the results stated in the corresponding paper; the results obtained after running their code has been reported in this work.
- One being traditional matrix completion (MC: matrix completion) [47] and
- Last one being a naive solution to our problem, available as an unpublished work (MCG: matrix completion on graphs). Of note, the Space complexity of MCG is *O*(*n*^4^) while that of MGRNNM is *O*(*n*^2^). [56])

All baselines designed for DTI problem are recent and are already compared against older methods.

We have performed 5 runs of 10-fold cross-validation (CV) for each of the algorithms under three cross-validation setting (CVS) [2]:

- CVS1/Pair prediction: random drug–target pairs are left out for testing set to be used in prediction. It is the conventional setting for validation and evaluation.
- CVS2/Drug prediction: the complete drug profiles are left out for the testing set. It tests the algorithm’s ability to predict interactions for novel drugs i.e. drugs for which no interaction information is available.
- CVS3/Target prediction: the complete target profiles are left out for the testing set. It tests the algorithm’s ability to predict interactions for novel targets.

In 10-fold CV, the given data was divided into 10 folds and out of those 10 folds, one was left out for testing whereas the remaining 9 folds were used as the training set. As the evaluation metrics, we have used AUC (Area under ROC curve) and AUPR (Area under the precision-recall curve). In biological drug discovery, AUPR is a practically more important metric since it penalizes high ranked false positive interactions much more than AUC. This is because those pairs would be biologically validated later in the drug discovery process.

### Preprocessing

Each of the drug and target similarity matrices were summed up to compute the combined similarity matrices $S_{d}^{COM}$ and $S_{d}^{COM}$ (Eq (17)). The combined similarity matrices were further sparsified by using p-nearest neighbor graph which is obtained by taking into account only the similarity values which correspond to the nearest neighbors for each drug/target. The usage of such a pre-processing, as shown by [37], helps learn a data manifold on or near to which the data is assumed to lie which, in turn, is expected to preserve the local geometries of the original data and hence give more accurate results.

$$\begin{array}{c} \begin{array}{c} \forall i,j\\ N_{ij}=\{\begin{array}{c} 1,j\in N_{p}\left(i\right)\&i\in N_{p}\left(j\right)\\ 0,j\notin N_{p}\left(i\right)\&i\notin N_{p}\left(j\right)\\ 0.5,else \end{array} \end{array} \end{array}$$

where *Np*(*i*) is the set of *p* nearest neighbors to drug *d*_*i*_. Similarity matrix sparsification is done by element-wise multiplying it with *N*_*ij*_. In the next step, the combined graph laplacian terms are computed. Also, instead of graph laplacians ($L_{d}^{COM}$ and $L_{t}^{COM}$), we have used normalized graph laplacians (${\left(D_{d}^{COM}\right)}^{-1/2}L_{d}^{COM}{\left(D_{d}^{COM}\right)}^{-1/2}$ and ${\left(D_{t}^{COM}\right)}^{-1/2}L_{t}^{COM}{\left(D_{t}^{COM}\right)}^{-1/2}$) instead as normalized graph Laplacians are known to perform better in many cases [57].

### Parameter settings

For setting the parameters of our algorithm, we performed cross-validation on the training set on the parameters *p*, λ, *μ*_1_, *μ*_2_, *ν*_1_, *ν*_2_ to find the best parameter combination for each dataset, under each cross-validation setting. As mentioned earlier, the individual laplacians or the similarities can be weighted unequally to give more or less emphasis on a specific type of similarity, we weigh the Cosine, Correlation and Jaccard similarities heavily (4 times) relative to Hamming similarity. This was done because hamming similarity showed the least improvement in prediction accuracy as compared to the other three similarities when taken into account along with standard similarities. For the other methods, we set the parameters to their optimal (which were found to be already optimal) in [2].

### Interaction prediction

Tables 2 and 3 show the AUPR results and the AUC results for the CVS1 cross validation setting respectively. The following tables (Tables 4, 5, 6 and 7) report the CVS2 and CVS3 validation setting results. The second column in each table shows the results of our algorithm when only the standard similarity matrices (*S*_*d*_: chemical structure similarity for drugs, *S*_*t*_: Genomic sequence similarity for target proteins) were used for prediction.

**Table 2:** AUPR results for interaction prediction under validation setting CVS1.

AUPR	MGRNNM	standard	MC	MCG	WGRMF	RLS_WNN	CMF	NRLMF	TMF
E	**0.9660** **(0.0006)**	0.9014 (0.0018)	0.7882 (0.0022)	0.7621 (0.0025)	0.8768 (0.0020)	0.8093 (0.0045)	0.8837 (0.0026)	0.8749 (0.0017)	0.7886 (0.0020)
IC	**0.9585** **(0.0013)**	0.9298 (0.0026)	0.8868 (0.0028)	0.8346 (0.0025)	0.9225 (0.0022)	0.8459 (0.0106)	0.9373 (0.0019)	0.8674 (0.0056)	0.8654 (0.0041)
GPCR	**0.8515** **(0.0033)**	0.7483 (0.0039)	0.6481 (0.0116)	0.5956 (0.0102)	0.7370 (0.0024)	0.6933 (0.0226)	0.7543 (0.0017)	0.7115 (0.0144)	0.6600 (0.0059)
NR	**0.8791** **(0.0019)**	0.6408 (0.0234)	0.3950 (0.0298)	0.4558 (0.0202)	0.6016 (0.0378)	0.7072 (0.0290)	0.6383 (0.0149)	0.7390 (0.312)	0.4248 (0.0163)
Average	**0.9138**	0.8051	0.6795	0.6620	0.7845	0.7639	0.8034	0.7982	0.6847

**Table 3:** AUC results for interaction prediction under validation setting CVS1.

AUC	MGRNNM	standard	MC	MCG	WGRMF	RLS_WNN	CMF	NRLMF	TMF
E	**0.9955** **(0.0003)**	0.9798 (0.0004)	0.8753 (0.0023)	0.9596 (0.0015)	0.9647 (0.0013)	0.9635 (0.0014)	0.9705 (0.0013)	0.9761 (0.0017)	0.8943 (0.0030)
IC	**0.9947** **(0.0004)**	0.9829 (0.0012)	0.9415 (0.0015)	0.9539 (0.0010)	0.9747 (0.0022)	0.9786 (0.0026)	0.9832 (0.0008)	0.9838 (0.0009)	0.9433 (0.0017)
GPCR	**0.9785** **(0.0020)**	0.9531 (0.0028)	0.8110 (0.0055)	0.8977 (0.0047)	0.9432 (0.0010)	0.9458 (0.0044)	0.9493 (0.0031)	0.9620 (0.0023)	0.8373 (0.0038)
NR	**0.9660** **(0.0056)**	0.9083 (0.0058)	0.5882 (0.0253)	0.8315 (0.0165)	0.8892 (0.0153)	0.9329 (0.0114)	0.8679 (0.0124)	0.9479 (0.0045)	0.5496 (0.0296)
Average	**0.9837**	0.9560	0.8040	0.9107	0.9429	0.9552	0.9427	0.9674	0.8061

**Table 4:** AUPR results for interaction prediction under validation setting CVS2.

AUPR	MGRNNM	standard	MC	MCG	WGRMF	RLS_WNN	CMF	NRLMF	TMF
E	**0.8603** **(0.0095)**	0.4089 (0.0104)	0.0114 (0.0005)	0.0457 (0.0008)	0.4019 (0.0128)	0.2409 (00272)	0.3848 (0.0094)	0.3582 (0.0101)	0.3748 (0.0113)
IC	**0.9026** **(0.0197)**	0.3650 (0.0178)	0.0473 (0.0035)	0.0925 (0.0013)	0.3666 (0.0169)	0.3090 (0.0200)	0.3538 (0.0137)	0.3414 (0.0148)	0.3371 (0.0112)
GPCR	**0.8538** **(0.0112)**	0.4175 (0.0076)	0.0404 (0.0017)	0.1091 (0.0044)	0.4247 (0.0113)	0.3463 (0.0106)	0.4059 (0.0104)	0.3671 (0.067)	0.3866 (0.0078)
NR	**0.8773** **(0.0125)**	0.5620 (0.0262)	0.1120 (0.0206)	0.2404 (0.0337)	0.5695 (0.0136)	0.5373 (0.0216)	0.5203 (0.0250)	0.5296 (0.0348)	0.4912 (0.0230)
Average	0.8735	0.4384	0.0528	0.1219	0.4407	0.3584	0.4162	0.3990	0.3974

**Table 5:** AUC results for interaction prediction under validation setting CVS2.

AUC	MGRNNM	standard	MC	MCG	WGRMF	RLS_WNN	CMF	NRLMF	TMF
E	**0.9460** **(0.0033)**	0.8260 (0.0108)	0.5060 (0.0090)	0.7413 (0.0118)	0.7982 (0.0144)	0.7755 (0.0093)	0.7952 (0.0110)	0.8151 (0.0062)	0.8204 (0.0111)
IC	**0.9714** **(0.0095)**	0.7913 (0.0090)	0.5512 (0.0034)	0.7196 (0.0071)	0.7902 (0.0149)	0.7669 (0.0140)	0.7576 (0.0125)	0.7881 (0.0140)	0.8030 (0.0184)
GPCR	**0.9567** **(0.0084)**	0.8805 (0.0024)	0.5855 (0.0039)	0.7745 (0.0027)	0.8800 (0.0025)	0.8524 (0.0072)	0.8067 (0.0067)	0.8841 (0.0054)	0.8452 (0.0044)
NR	**0.9533** **(0.0127)**	0.8452 (0.0215)	0.5294 (0.0200)	0.6992 (0.0244)	0.8615 (0.0244)	0.8390 (0.0261)	0.8124 (0.0228)	0.8804 (0.0179)	0.8435 (0.0225)
Average	0.9568	0.8357	0.5430	0.7337	0.8325	0.8085	0.7930	0.8419	0.8280

**Table 6:** AUPR results for interaction prediction under validation setting CVS3.

AUPR	MGRNNM	standard	MC	MCG	WGRMF	RLS_WNN	CMF	NRLMF	TMF
E	**0.9041** **(0.0125)**	0.8087 (0.0156)	0.0124 (0.0005)	0.0691 (0.0009)	0.8070 (0.0185)	0.5465 (0.0144)	0.7808 (0.0131)	0.8112 (0.0166)	0.8000 (0.0167)
IC	**0.9029** **(0.0024)**	0.8079 (0.0096)	0.0421 (0.0043)	0.2256 (0.0038)	0.8128 (0.0069)	0.7437 (0.0088)	0.7786 (0.0108)	0.7753 (0.0072)	0.7893 (0.0073)
GPCR	**0.7228** **(0.0323)**	0.5963 (0.0336)	0.0549 (0.0105)	0.1061 (0.0027)	0.6093 (0.0314)	0.5397 (0.0193)	0.5989 (0.0323)	0.5515 (0.0234)	0.6001 (0.0243)
NR	**0.5418** **(0.0309)**	0.4356 (0.0177)	0.0850 (0.0227)	0.2669 (0.0288)	0.4643 (0.0183)	0.4907 (0.0326)	0.4774 (0.0173)	0.5207 (0.0247)	0.4709 (0.0256)
Average	0.7679	0.6621	0.0486	0.1669	0.6734	0.5801	0.6589	0.6646	0.6650

**Table 7:** AUC results for interaction prediction under validation setting CVS3.

AUC	MGRNNM	standard	MC	MCG	WGRMF	RLS_WNN	CMF	NRLMF	TMF
E	**0.9683** **(0.0043)**	0.9246 (0.0091)	0.5234 (0.0057)	0.8065 (0.0012)	0.9338 (0.0071)	0.9067 (0.0105)	0.9272 (0.0050)	0.9465 (0.0052)	0.9436 (0.0072)
IC	0.9541 (0.0019)	0.9346 (0.0041)	0.4724 (0.0065)	0.7871 (0.0069)	0.9460 (0.0034)	0.9286 (0.0046)	0.9368 (0.0032)	**0.9587** **(0.0027)**	0.9476 (0.0042)
GPCR	0.8975 (0.0093)	0.8798 (0.0134)	0.5683 (0.0310)	0.6289 (0.0151)	0.8892 (0.0110)	0.8694 (0.0146)	0.8966 (0.0073)	**0.9205** **(0.0052)**	0.8735 (0.0160)
NR	0.7502 (0.0285)	0.7263 (0.0211)	0.3767 (0.0204)	0.6522 (0.0297)	0.7967 (0.0132)	0.8124 (0.0202)	0.8373 (0.0083)	**0.8613** **(0.0097)**	0.8407 (0.0202)
Average	0.8909	0.8618	0.4575	0.7486	0.8922	0.8826	0.9004	0.9217	0.9013

The results clearly show that the interaction prediction in MGRNNM, not only shows great improvement on incorporation of new similarity types but also outperforms all the state-of-the-art prediction methods in terms of AUC and AUPR evaluation metrics in almost all test cases except in CVS3 (where the difference between AUPR obtained by the best-performing method: TMF and MGRNM is not much).

The degradation in performance in CVS3 setting can be attributed to the comparatively unstable results obtained for mainly NR dataset. This can be due to its excessively small size, as also concluded by [58].

It is also observed that inference of MGRNNM under CVS1 is always better than in CVS2/CVS3 because novel drugs/target proteins have no interaction available and hence CVS1 validation setting provides more information in the training data.

If we compare the performance of MGRNNM under CVS2 and CVS3 in all the datasets, an important factor which influence the results in these two cross-validation settings is the “drug to target ration” (say DTR). DTR for NR, GPCR, IC and E datasets are 54:26, 223:95, 105:102 and 445:664 respectively. Since, more information is the prior condition to achieve better inferences, performance under CVS2 should be better than in CVS3 for NR and GPCR datasets, performance under CVS2 and CVS3 should be similar for IC dataset and performance under CVS3 should be better than in CVS2. The results from MGRNNM perfectly follow this trend.

**Fig 2:**
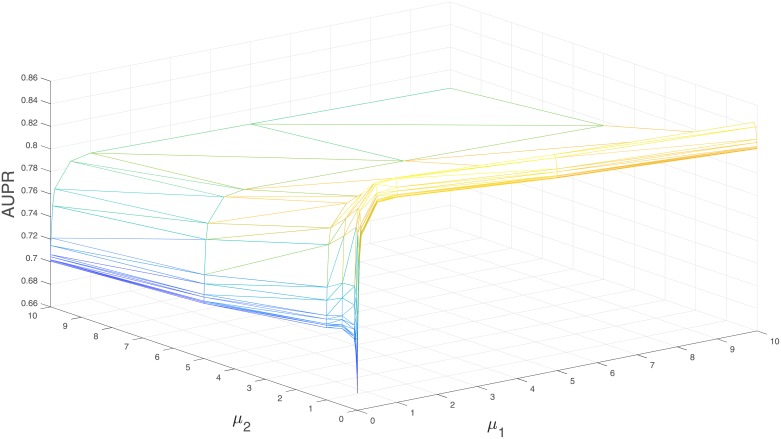
Three-dimensional mesh depicting the variation of AUPR with the parameters *μ*_1_ and *μ*_2_ for drug-target interaction prediction using MGRNNM.

We also analyze the performance of MGRNNM with the two regularization parameters *μ*_1_ and *μ*_2_, which govern the incorporation of the two graph laplacian terms in our algorithm. As an example, Fig 2 shows how these two parameters affect the prediction in case of GPCR dataset under cross-validation setting CVS1. When *μ*_1_ and *μ*_2_ are close to zero, the value of AUPR is 0.67; whereas when *μ*_1_ and *μ*_2_ gradually increase, the value of AUPR improves (more steep increase with *μ*_1_ than with *μ*_2_), achieving the best value (0.85) at *μ*_1_ = 0.5 and *μ*_2_ = 0.1 validating the effectiveness of multiple graph Laplacian components.

### Validation of multiple similarities

To precisely analyze the consequence of multiple similarities incorporation, we observed the mean AUPR for several cases:

- standard: When only the standard similarity matrices (*S*_*d*_: chemical structure similarity for drugs, *S*_*t*_: Genomic sequence similarity for target proteins) were used for prediction.
- standard+Cosine: When Cosine similarity between each pair of drugs/targets ($S_{d}^{cos}$, $S_{t}^{cos}$) was taken into account along with standard similarities.
- standard+Correlation: When Pearson’s linear Correlation between each pair of drugs/targets ($S_{d}^{cor}$, $S_{t}^{cor}$) was taken into account along with standard similarities.
- standard+Hamming: When Hamming similarity between each pair of drugs/targets ($S_{d}^{ham}$, $S_{t}^{ham}$) was taken into account along with standard similarities.
- standard+Jaccard: When Jaccard similarity between each pair of drugs/targets ($S_{d}^{jac}$, $S_{t}^{jac}$) was taken into account along with standard similarities.
- COMBINED: When all five similarity types between each pair of drugs/targets ($S_{d}^{COM}$, $S_{t}^{COM}$) were taken into account.

**Fig 3:**
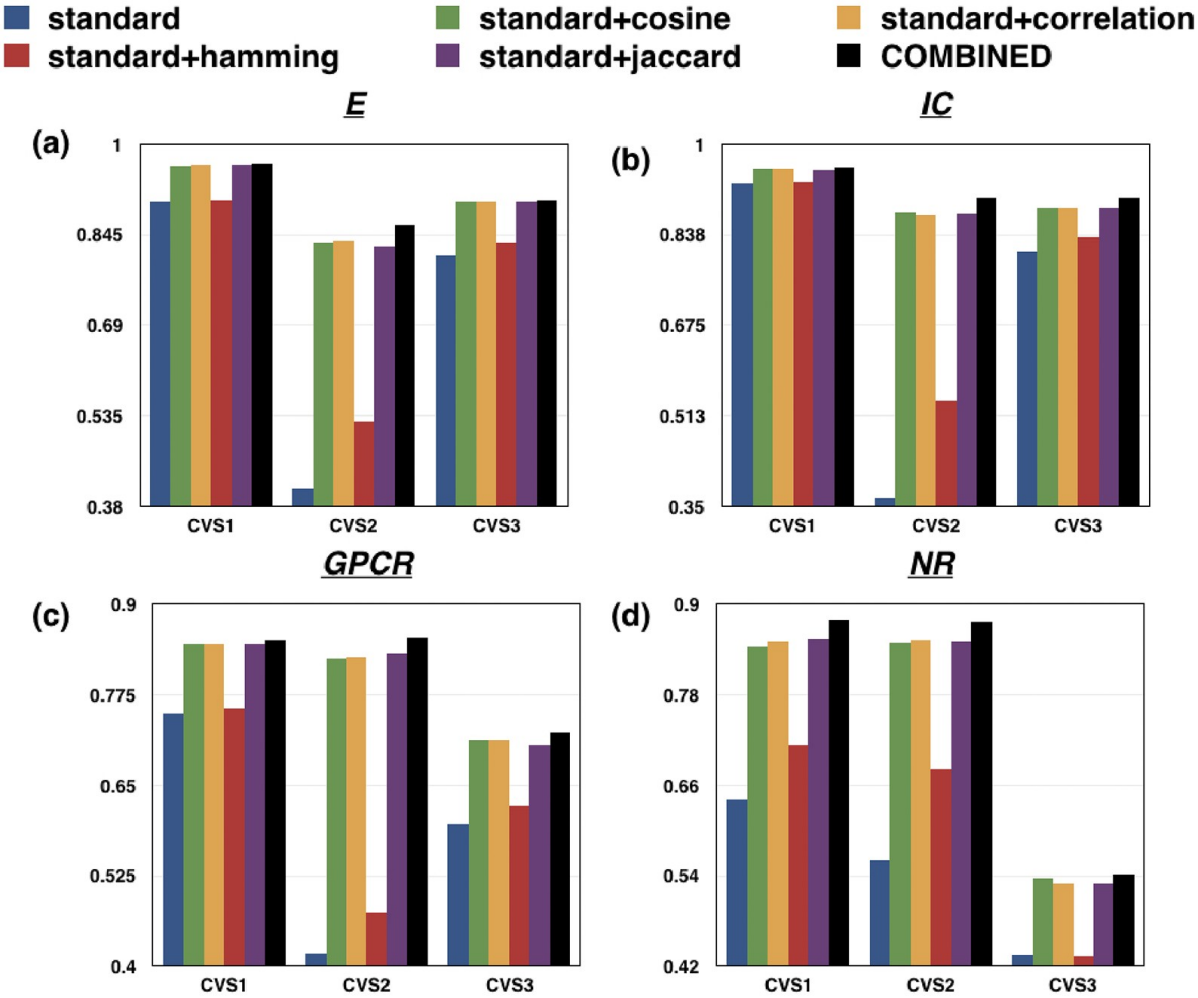
Bar plots depicting that incorporating all the similarities for drugs and targets for prediction task yields best results for every dataset (a) E (b) IC (c) GPCR and (d) NR under the three cross-validation settings in comparison to the cases where each type of similarity was considered separately. Here, “standard” represents the case when only the chemical structure similarity for drugs and genomic sequence similarity for targets were taken into account and “COMBINED” refers to the use case where all the similarity matrices (standard similarity, Cosine similarity, Correlation, Hamming similarity and Jaccard similarity) were considered.

The analysis was carried out for every dataset under all the three cross-validation settings. Fig 3 clearly depicts that incorporating all the similarities for drugs and targets for prediction task yields the best results.

## Conclusion

Drug-target interaction prediction is a crucial task in genomic drug discovery. Many computational techniques have been proposed in the literature. In this work, we presented a novel chemogenomic approach for predicting the drug-target interactions, MGRNNM (Multi-Graph regularized Nuclear Norm Minimization). It is a graph regularized version of the traditional Nuclear Norm Minimization algorithm which incorporates multiple Graph Laplacians over the drugs and targets into the framework for an improved interaction prediction. The algorithm is generic and can be used for prediction in protein-protein interaction [59], RNA-RNA interaction [60], etc.

The evaluation was performed using three different cross-validation settings, namely CVS1 (random drug-target pairs left out), CVS2 (entire drug profile left out) and CVS3 (entire target profile left out) to compare our method with 5 other state-of-the-art methods (three specifically designed for DTI prediction). In almost all of the test cases, our algorithm shows the best performance, outperforming the baselines. This work can be extended by accounting for more types of drug and target similarities which could be either chemically/biologically driven or obtained from the metadata itself to improve the prediction accuracy even further.

## Supporting information

S1 File Supplementary file showing the experimental results on the analysis of the improvements achieved by the MGRNNM algorithm and statistical significance of the improvement of MGRNNM over the other methods. (PDF)
