# Supplementary material for: Drug-target interaction prediction using Multi Graph Regularized Nuclear Norm Minimization
Source: PLoS One. 2020 Jan 16;15(1):e0226484. doi: 10.1371/journal.pone.0226484 (PMC6964976; doi:10.1371/journal.pone.0226484)
Supplement: S1 File — (PDF) [file pone.0226484.s001.pdf]

Supplementary

Drug-Target Interaction prediction using Multi Graph Regularized Nuclear Norm Minimization

Aanchal Mongia[1], Angshul Majumdar[2*]

1 Dept. of Computer Science and Engineering/IIIT-Delhi/Delhi-110020/India
2 Dept. of Electronics and Communications Engineering/IIIT-Delhi/Delhi-110020/India

* Corresponding Author

Email address: {aanchalm, angshul}@iiitd.ac.in

1. Ablation Study

To analyse the improvements achieved by the MGRNNM algorithm, we have carried out an ablation study. In addition to the similarity metrics shown in Figure 2, We have conducted experiments on the newly introduced individual similarity metrics separately and the combined similarity obtained by summing up all kinds of similarities. The results have been shown in Supplementary Figure 1 below, depicting that the combined one yield better prediction than any of the individual ones.

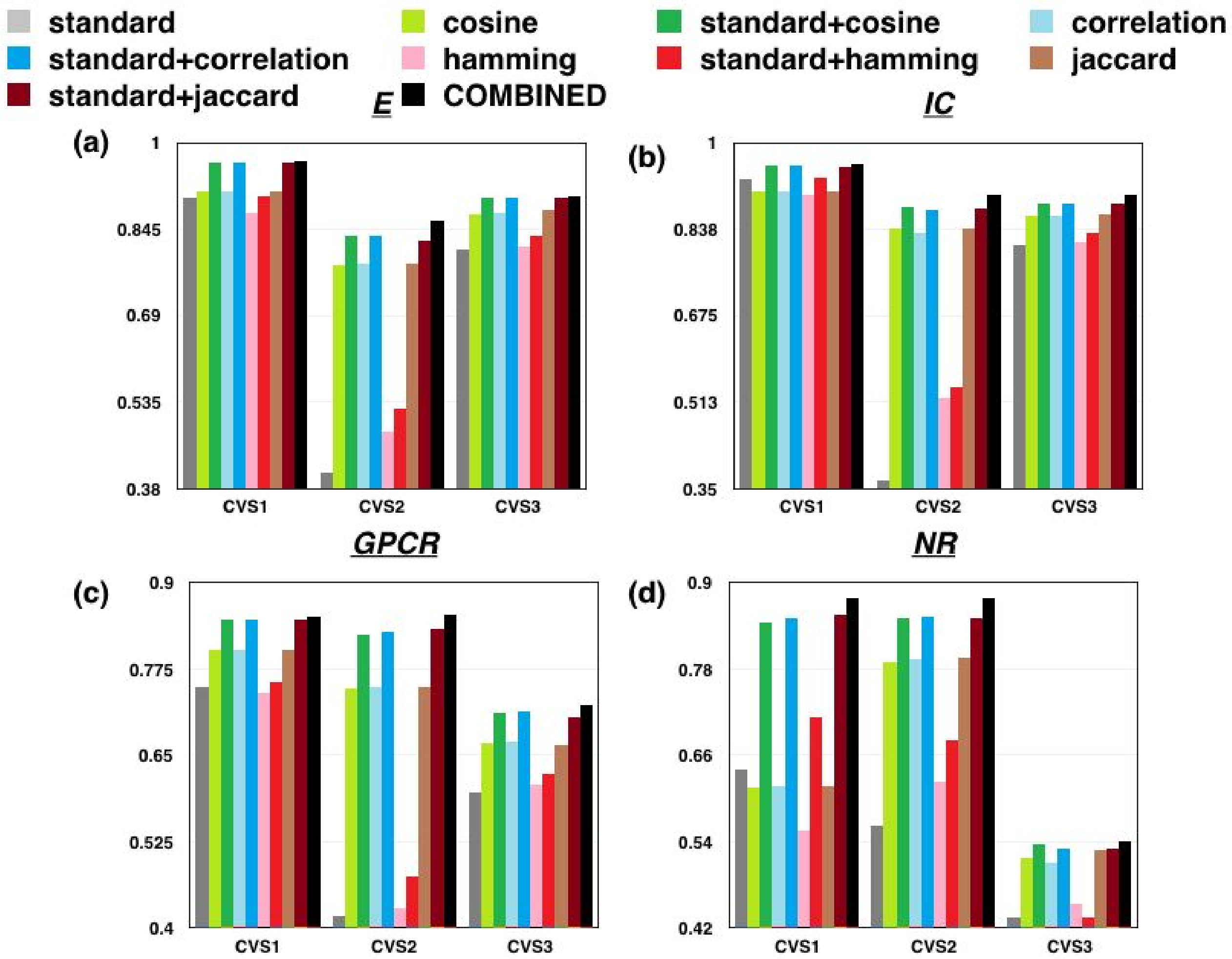

Supplementary Figure 1: Bar plots depicting that incorporating all the similarities for drugs and targets for prediction task yields best results for every dataset (a) E (b) IC (c) GPCR and (d) NR under the three cross-validation settings in comparison to the cases where each type of similarity was considered separately.

2. Statistical Testing

We have conducted a paired t-test to analyse the statistical significance of the improvement of MGRNNM over the other methods in terms of AUPR (over 10 runs of 10 fold cross validation).

The p-values obtained after performing t-test are reported in **Supplementary Tables 1- 8**. It can be observed that the improvement achieved by MGRNNM is statistically significant (5% significance level) in all test cases for all the baseline methods except RLS_WNN and NRLMF where this is not the case for 1 in 12 test cases each.

**STANDARD**	E	IC	GPCR	NR
CVS1	1.30e-16	3.86e-14	7.69e-17	5.65e-12
CVS2	7.13e-15	1.56e-13	5.43e-17	2.97e-09
CVS3	5.43e-14	5.29e-10	2.83e-07	7.73e-05

Supplementary Table 1: p-values obtained from paired-t-test conducted on AUPR values from MGRNNM and Standard approach (GR-NNM with standard similarities only)

**MC**	E	IC	GPCR	NR
CVS1	7.32e-18	9.19e-15	1.51e-14	1.51e-12
CVS2	2.55e-18	1.55e-17	1.96e-18	6.58e-14
CVS3	3.14e-19	4.33e-20	9.20e-15	4.47e-11

Supplementary Table 2:p-values obtained from paired-t-test conducted on AUPR values from MGRNNM and MC (Matrix Completion)

**MCG**	E	IC	GPCR	NR
CVS1	1.04e-17	2.32e-16	2.47e-14	8.07e-14
CVS2	6.79e-18	4.74e-17	2.62e-18	2.46e-11
CVS3	4.97e-19	1.66e-18	1.45e-13	7.36e-10

Supplementary Table 3: p-values obtained from paired-t-test conducted on AUPR values from MGRNNM and MCG (Matrix Completion on Graphs)

**WGRMF**	E	IC	GPCR	NR
CVS1	1.46e-17	3.96e-14	4.98e-17	2.34e-10
CVS2	2.39e-16	1.69e-13	3.42e-16	3.78e-11
CVS3	1.72e-11	1.23e-08	7.32e-06	0.0198

Supplementary Table 4: p-values obtained from paired-t-test conducted on AUPR values from MGRNNM and GRMF (Weighted Graph regularized Matrix Factorization)

**RLS_WNN**	E	IC	GPCR	NR
CVS1	3.34e-15	1.48e-11	5.27e-11	3.07e-08
CVS2	1.52e-13	2.02e-13	5.16e-16	6.82e-10
CVS3	4.46e-15	2.10e-10	1.43e-07	0.1443

Supplementary Table 5: p-values obtained from paired-t-test conducted on AUPR values from MGRNNM and RLS-WNN (Regularized Least square Nearest neighbor profile)

**CMF**	E	IC	GPCR	NR
CVS1	1.92e-16	1.26e-12	1.37e-15	8.75e-10
CVS2	3.40e-15	2.32e-13	1.45e-15	1.05e-10
CVS3	2.96e-13	1.76e-09	7.69e-06	0.0091

Supplementary Table 6: p-values obtained from paired-t-test conducted on AUPR values from MGRNNM and CMF (Collaborative Matrix factorization)

**TMF**	E	IC	GPCR	NR
CVS1	2.13e-18	1.49e-13	1.77e-15	6.90e-14
CVS2	3.34e-15	5.60e-14	1.30e-15	5.74e-12
CVS3	6.00e-12	1.05e-10	5.78e-06	0.0033

Supplementary Table 7: p-values obtained from paired-t-test conducted on AUPR values from MGRNNM and TMF (Triple Matrix Factorization)

**NRLMF**	E	IC	GPCR	NR
CVS1	1.68e-16	3.07e-12	2.45e-11	1.01e-07
CVS2	2.56e-15	7.10e-14	1.41e-17	1.35e-10
CVS3	1.60e-10	7.99e-10	6.73e-07	0.1566

Supplementary Table 8: p-values obtained from paired-t-test conducted on AUPR values from MGRNNM and NRLMF (Neighborhood Regularized Logistic Matrix Factorization)
